# STX17: an ancient SNARE protein whose roles have not been conserved

**DOI:** 10.1080/27694127.2022.2033056

**Published:** 2022-03-22

**Authors:** Shun Kato, Mitsuo Tagaya

**Affiliations:** School of Life Sciences, Tokyo University of Pharmacy and Life Sciences, Hachioji,Tokyo 192-0392, Japan

**Keywords:** Autophagy, evolution, lipid droplet, mitochondrial fission, syntaxin 17

## Abstract

Mammalian STX17 (syntaxin 17) plays different cellular roles, including in mitochondrial fission, lipid droplet expansion and macroautophagy/autophagy, depending on the nutritional status. STX17 has a long C-terminal hydrophobic domain (CHD) with a hairpin-like structure, flanked by a basic amino acid-enriched C-terminal tail (C-tail). STX17 is present in a wide variety of eukaryotes, but has been lost in several lineages during evolution. Moreover, the structure of its C-tail remarkably varies, although the CHD is highly conserved. Recently, we compared the localization and function of fly and nematode Syx17/SYX-17 proteins expressed in mammalian cells with that of human STX17. Fly Syx17 expressed in mammalian cells localizes almost exclusively to the cytosol and translocates to autophagosomes upon starvation, whereas nematode SYX-17 is mainly distributed to mitochondria and promotes mitochondrial fission, but does not participate in autophagy. In vivo experiments showed that fly and nematode STX17 homologs are not involved in mitochondrial fission and autophagy, respectively. These results provide important insights into the localization and function of STX17, which acts as a molecular sensor for different nutritional conditions.

Mammalian STX17 (syntaxin 17) has been reported to be involved in two stages of autophagy, i.e., autophagosome formation, by recruiting through interaction with ATG14 the class III phosphatidylinositol 3-kinase complex to the mitochondria-associated membranes (MAMs), and autophagosome-endolysosome fusion by pairing with SNAP29 (synaptosomal-associated protein 29) and VAMP8 (vesicle-associated membrane protein 8). In addition, this SNARE protein plays roles in mitochondrial fission under nutrient-rich conditions and lipid droplet expansion under conditions of excess nutrients (presence of large amounts of oleic acid), by interacting with DNM1L/DRP1 (dynamin 1 like) and ACSL3 (acyl-CoA synthetase long chain family member 3), respectively. Thus, STX17 can serve as a nutrient sensor and mediate various cellular processes as a scaffold protein as well as a membrane fusion effector.

STX17 is one of the six ancient eukaryotic Qa-soluble N-ethylmaleimide-sensitive factor attachment protein receptor (SNARE) proteins, but has been lost in several lineages, including yeast, during evolution. While conventional syntaxins have a transmembrane domain consisting of 17-24 amino acids after the SNARE motif, STX17 possesses a long C-terminal hydrophobic domain (CHD) of 44 amino acids, containing two hydrophobic segments separated by Lys254, which is followed by a C-terminal tail (C-tail). The CHD is highly conserved across species (Figure 1, upper panel), and likely forms a hairpin-like structure with Lys254 possibly facing the cytosol. In contrast, the amino acid sequence of the C-tail varies greatly. Human and nematode STX17/SYX-17 have many positively charged amino acids, whereas fly Syx17 has many negatively charged residues.

**Figure 1.**
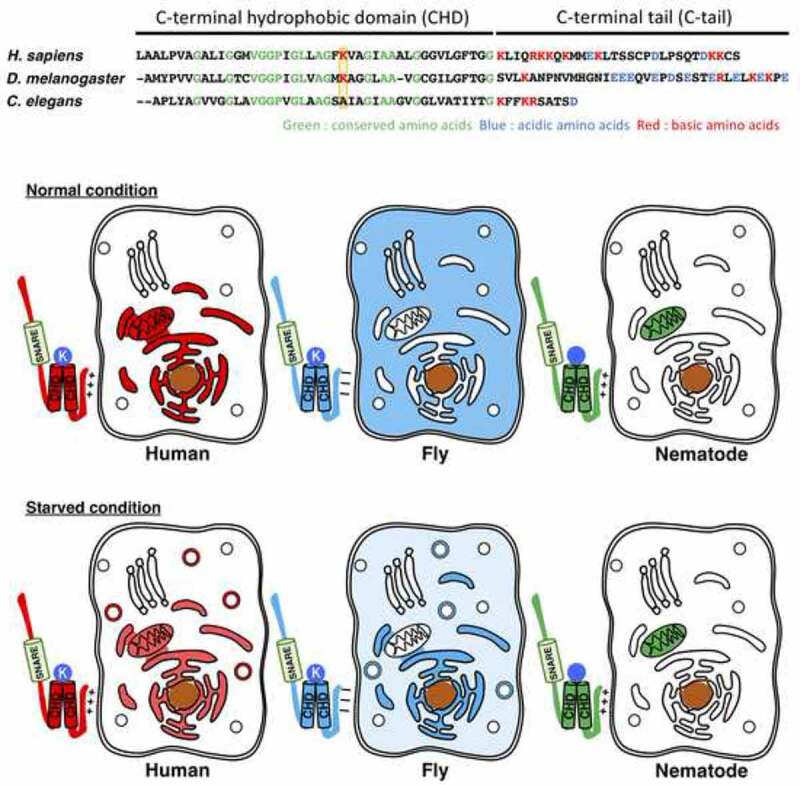
Sequence and localization of human, fly and nematode STX17 homologs.Upper panel. Comparison of the amino acid sequence of the CHD and C-tail of human, fly and nematode STX17 homologs. Identical, basic, and acidic amino acids are highlighted in green, red, and light blue, respectively. Lys254 in human and its corresponding amino acid in fly (Lys248) and nematodes (Ala222) are boxed in orange. Middle panel. Localization of STX17 proteins under nutrient-rich conditions. Human STX17 (red) localizes to the endoplasmic reticulum, MAMs and mitochondria. Fly Syx17 (light blue) is predominantly present in the cytosol, whereas nematode SYX-17 (green) mainly localizes to mitochondria. Lower panel. Localization of STX17 proteins under starvation conditions. Upon starvation, human and fly STX17/Syx17 translocate onto autophagosomes, whereas nematode SYX-17 remains localized to the mitochondria. Of note, the STX17 homologs localize to the surface of the indicated membranes.

In our recent study [1], we examined the localization and function of fly and nematode STX17 homologs in mammalian cells. Human and nematode STX17/SYX-17 proteins with their positively charged C-tail, are distributed to the MAMs and/or mitochondria, whereas fly Syx17 with its negatively charged C-tail almost exclusively localizes to the cytosol (Figure 1, middle panel). Upon nutrient starvation, fly Syx17 as well as human STX17, translocates onto autophagosomes, consistent with the previous report that human and fly STX17/Syx17 participate in autophagosome-endolysosome fusion. Nematode SYX-17, in contrast, remains associated with mitochondria (Figure 1, lower). Chimeric analysis revealed that the C-tail is the determinant for localization and membrane association. Removing three positive charges from the C-tail of nematode SYX-17, causes the resulting mutant to be predominantly present in the cytosol. Lys254 in human STX17 also contributes to localization. Replacement of Lys254 in the human protein and the corresponding Lys248 in fly Syx17 with an Ala residue, abrogates autophagosome localization. Replacement of Ala222 in nematode SYX-17, corresponding to Lys254 in the human protein, with a Lys did not confer to the resulting protein the capacity to be recruited onto autophagosomes, suggesting that this Lys residue is essential but not sufficient for SYX-17 targeting onto autophagosomes.

Depletion of STX17 in mammalian cells causes a defect in mitochondrial fission, autophagosome formation, and lipid droplet expansion. Expression of nematode SYX-17 in these cells can rescue the mitochondrial fission defect, but not the impairment in autophagosome formation. In contrast, expression of fly Syx17 can compensate for the defect in autophagosome formation, but not in mitochondrial fission. Consistent with these results, nematode SYX-17 interacts with DNM1L, but not ATG14, whereas fly Syx17 binds to ATG14, but not DNM1L. Moreover, both fly and nematode STX17 homologs interact with ACSL3 and rescue the defect in lipid droplet expansion of STX17-depleted mammalian cells. The results of the in vivo studies are largely consistent with these findings: knockout of fly *Syx17* has no effect on mitochondrial length in the indirect flight muscle, and SYX-17 mutation in nematode does not affect allophagy, a selective type of autophagy that degrades paternal mitochondria during fertilization.

Why have the functions of STX17 not been conserved during evolution, although it is one of six ancient eukaryotic Qa-SNARE proteins? This may be due to the emergence of other scaffolding proteins during evolution and/or the evolution of proteins involved in mitochondrial fission and autophagosome formation, which may have reduced the need for STX17. As a result, STX17 homologs in nematodes and flies might have lost its ability to participate in autophagosome formation and mitochondrial fission, respectively. The fact that yeast does not possess a STX17 ortholog is probably due to the presence of a different set of SNAREs involved in autophagy. In yeast, Ykt6, Vam3, Vti1 and Vam7 are the SNARE proteins that have been implicated in autophagosome-vacuole fusion. YKT6, the metazoan counterpart of Ykt6, also participates in autophagy in mammalian cells.

One important question that remains to be elucidated is how does STX17 translocate onto autophagosomal membranes. In fly, post-translational modification such as phosphorylation/dephosphorylation or proteins that bind to Syx17, may support translocation of Syx17 from the cytosol onto membranes. In mammalian cells, the hairpin-like structure of the CHD may be important for STX17 to leap from the MAMs and/or mitochondria, onto autophagosomes. A previous study has shown that CYB5 (cytochrome b5), which has a hairpin-like structure, also translocates from mitochondria to autophagosomes, probably via the MAMs, upon starvation. Elucidation of the mechanism underlying translocation of STX17 onto autophagosomes may provide insights into the role of the unique, species-specific C-tail structure of STX17 proteins.
